# Supplementary material for: Modeling glioblastoma heterogeneity as a dynamic network of cell states
Source: Mol Syst Biol. 2021 Sep 16;17(9):e10105. doi: 10.15252/msb.202010105 (PMC8444284; doi:10.15252/msb.202010105)
Supplement: Supplementary file 6 — Source Data for Figure 5 [file MSB-17-e10105-s004.zip › Figure5A_sourcedata/GSEA_3017/nosigdb_stateC.GseaPreranked.1622795002785/IVY_CTPAN.html]

Details for gene set IVY\_CTPAN[GSEA]

|  || Dataset | state13017 |
| Phenotype | NoPhenotypeAvailable |
| Upregulated in class | na\_neg |
| GeneSet | IVY\_CTPAN |
| Enrichment Score (ES) | -0.519805 |
| Normalized Enrichment Score (NES) | -2.6617374 |
| Nominal p-value | 0.0 |
| FDR q-value | 0.0 |
| FWER p-Value | 0.0 |
Table: GSEA Results Summary

  

Fig 1: Enrichment plot: IVY\_CTPAN      
 Profile of the Running ES Score & Positions of GeneSet Members on the Rank Ordered List

  

| PROBE | GENE SYMBOL | GENE\_TITLE | RANK IN GENE LIST | RANK METRIC SCORE | RUNNING ES | CORE ENRICHMENT || 1 | RND3 |  |  | 2 | 0.713 | 0.0418 | No |
| 2 | IGF2BP2 |  |  | 231 | -0.260 | -0.3466 | No |
| 3 | SOCS3 |  |  | 274 | -0.270 | -0.4041 | No |
| 4 | NAMPT |  |  | 306 | -0.280 | -0.4413 | No |
| 5 | TMEM158 |  |  | 315 | -0.284 | -0.4375 | No |
| 6 | SERPINE1 |  |  | 323 | -0.286 | -0.4318 | No |
| 7 | FCGR2B |  |  | 326 | -0.286 | -0.4171 | No |
| 8 | PPP1R15A |  |  | 342 | -0.293 | -0.4251 | No |
| 9 | PDPN |  |  | 372 | -0.307 | -0.4571 | No |
| 10 | FOS |  |  | 377 | -0.309 | -0.4445 | No |
| 11 | SLC2A1 |  |  | 383 | -0.311 | -0.4336 | No |
| 12 | IGFBP5 |  |  | 384 | -0.311 | -0.4138 | No |
| 13 | GADD45A |  |  | 405 | -0.323 | -0.4288 | No |
| 14 | SLC2A3 |  |  | 422 | -0.336 | -0.4358 | No |
| 15 | VEGFA |  |  | 436 | -0.345 | -0.4370 | No |
| 16 | LGALS3 |  |  | 471 | -0.374 | -0.4736 | No |
| 17 | LDHA |  |  | 498 | -0.412 | -0.4936 | Yes |
| 18 | TGIF1 |  |  | 502 | -0.421 | -0.4721 | Yes |
| 19 | JUN |  |  | 503 | -0.425 | -0.4451 | Yes |
| 20 | SYTL2 |  |  | 513 | -0.439 | -0.4332 | Yes |
| 21 | IRS2 |  |  | 516 | -0.444 | -0.4085 | Yes |
| 22 | CEBPD |  |  | 523 | -0.455 | -0.3902 | Yes |
| 23 | IGFBP3 |  |  | 538 | -0.493 | -0.3837 | Yes |
| 24 | IL1RAP |  |  | 558 | -0.544 | -0.3828 | Yes |
| 25 | TNC |  |  | 564 | -0.572 | -0.3554 | Yes |
| 26 | MT2A |  |  | 566 | -0.575 | -0.3206 | Yes |
| 27 | CD44 |  |  | 575 | -0.659 | -0.2929 | Yes |
| 28 | SPP1 |  |  | 577 | -0.674 | -0.2518 | Yes |
| 29 | VIM |  |  | 578 | -0.674 | -0.2089 | Yes |
| 30 | S100A10 |  |  | 582 | -0.691 | -0.1702 | Yes |
| 31 | CYR61 |  |  | 584 | -0.714 | -0.1266 | Yes |
| 32 | CA12 |  |  | 586 | -0.717 | -0.0828 | Yes |
| 33 | HAS2 |  |  | 587 | -0.740 | -0.0357 | Yes |
| 34 | EMP1 |  |  | 589 | -0.785 | 0.0124 | Yes |
Table: GSEA details [plain text format]

  

Fig 2: IVY\_CTPAN: Random ES distribution      
 Gene set null distribution of ES for **IVY\_CTPAN**
